# Impact of metabolic-associated fatty liver disease on the cholesterol efflux capacity of high-density lipoproteins in adolescents with type 2 diabetes

**DOI:** 10.3389/fped.2024.1462406

**Published:** 2024-12-24

**Authors:** José Antonio Orozco Morales, Aída Xochitl Medina Urrutia, Margarita Torres Tamayo, Juan Reyes Barrera, Esteban Jorge Galarza, Juan Gabriel Juárez Rojas, Pilar Dies Suarez, Nahum Méndez Sánchez, Luis Enrique Díaz Orozco, Lubia Velázquez-López, Patricia Medina Bravo

**Affiliations:** ^1^ Department of Endocrinology, Hospital Infantil de México Federico Gómez, Mexico City, Mexico; ^2^ Odontological and Health Sciences, Universidad Nacional Autónoma de México, Mexico City, Mexico; ^3^ Department of Endocrinology, Instituto Nacional de Cardiología Ignacio Chávez, Mexico City, Mexico; ^4^ Supervision Coordination of IMSS-BIENESTAR, Mexican Social Security Institute (Instituto Mexicano del Seguro Social, IMSS), Mexico City, Mexico; ^5^ Department of Imaging, Hospital Infantil de México Federico Gómez, Mexico City, Mexico; ^6^ Faculty of Medicine, Universidad Nacional Autónoma de México, Mexico City, Mexico; ^7^ Liver Research Unit, Fundación Clínica Médica Sur, Mexico City, Mexico; ^8^ Clinical Epidemiology Research Unit, Hospital Carlos Mac Gregor Sánchez Navarro, Mexican Social Security Institute (Instituto Mexicano del Seguro Social, IMSS), Mexico City, Mexico

**Keywords:** diabetes, MAFLD, adolescents, HDL, cholesterol-efflux, lipids

## Abstract

**Context:**

Type 2 diabetes (DM2) is an emerging disease in the pediatric population. DM2 is associated with metabolic-associated fatty liver disease (MAFLD). High-density lipoproteins (HDLs) are lipoproteins that are believed to have atheroprotective properties that reduce the risk of cardiovascular disease (CVD). Current evidence suggests that the physicochemical and functional features of HDLs may play a key role in the pathogenesis of atherosclerosis.

**Objective:**

We aimed to assess the impact of MAFLD on cholesterol efflux capacity (CEC) in adolescents with DM2.

**Design:**

A cross-sectional study.

**Setting:**

Attention clinic for Children with Diabetes of the Hospital Infantil de México Federico Gómez.

**Patients or other participants:**

This study included a total of 70 adolescents, 47 of which had DM2 and 23 were healthy individuals.

**Interventions:**

The presence of MAFLD was determined by MR spectroscopy with proton density fat fraction. We compared the distribution of HDL subtypes (HDL2b, HDL2a, HDL3a, HDL3b, and HDL3c) and the chemical composition of HDLs (total protein, triglycerides, phospholipids, cholesteryl esters, and free cholesterol). HDL functionality was determined by the CEC, measuring the fluorescent cholesterol efflux from J774 macrophage cells.

**Main outcome measures:**

We were expecting to observe a decrease in HDL efflux capacity in adolescents with type 2 diabetes and MAFLD.

**Results:**

In our study, we observed a prevalence of MAFLD in 66% of adolescents with DM2, similar to that reported in other international studies (60%–80%). In the population with DM2 and MAFLD, we did not observe a decrease in CEC. Initially we found a slight elevation of CEC in adolescents with DM2, however, with the increase in liver fat, a little decrease is observed, which could explain a probable metabolic phenomenon, since the physicochemical composition and distribution of the particles is associated with the percentage of liver fat. A positive correlation between the percentage of liver fat and the concentration of HDL2b (*p* = 0.011), HDL2a (*p* = 0.014) and average particle size (*p* = 0.011) and the proportion of triglycerides inside the particles (*p* = 0.007). Likewise, negative correlation were found with the percentage of liver fat, cholesterol esters (*p* = 0.010) and free cholesterol of the particles (*p* < 0.001). We observed a positive correlation between CEC and the percentage of triglycerides (*p* = 0.007), and a negative correlation with the percentage of cholesterol esters (*p* = 0.05) inside the HDL's particles.

**Conclusions:**

In this group of adolescents with DM2, the presence of MAFLD was not associated with CEC; however, it is associated with abnormalities in the distribution and lipid composition of HDL particles. The momentum generated by the original proposal for MAFLD in the adult population and following the recommendations for pediatric MAFLD will be a step forward in helping to study the impact of MAFLD on the atheroprotective properties of HDL in the pediatric population.

## 1 Introduction

Type 2 diabetes (DM2) is an emerging disease in the pediatric population (1). Twenty years ago, DM2 accounted for less than 3% of all cases of new-onset diabetes observed in adolescents; however, it now accounts for 45% of cases (2). The occurrence of DM2 in adolescents poses a new challenge for health systems because patients with early-onset DM2 present a more aggressive evolution of the disease with a higher incidence of complications at younger ages (3, 4). DM2 patients have a higher risk for cardiovascular disease (CVD) and associated clinical complications (5, 6). Up to 97% of DM2 adolescents are overweight or obese (7), and they therefore tend to develop associated comorbidities that include hypertension, dyslipidemia, non-alcoholic fatty liver disease (NAFLD), and polycystic ovary syndrome (8). Till date, data on morbidity associated with DM2 in the pediatric population are scarce (9).

The prevalence of NAFLD has more than doubled during the past 20 years in children and adolescents in the USA, and has become the leading cause of chronic liver disease worldwide (10). An analysis of a group of published studies yielded a global NAFLD prevalence of 7.6% (95% confidence interval: 5.5%–10.3%) in the overall pediatric population and a global NAFLD prevalence of 34.2% (95% confidence interval: 27.8%–41.2%) among obese children (11, 12). Regarding race, NAFLD prevalence is higher in Hispanic populations, followed by non-Hispanic white people and African Americans; in a subgroup analysis, the Mexican population had the highest prevalence (13). In 2020, an international expert consensus panel suggested a redefinition of adult fatty liver disease associated with metabolic dysregulation; this redefinition included an update of the negative term, NAFLD, to the term metabolic (dysfunction)-associated fatty liver disease (MAFLD) and the introduction of a simplified and easily applicable set of positive criteria for diagnosis (14, 15). The new definition of MAFLD has been proposed and accepted by an international consensus of experts in the pediatric population (16); including a group of pediatric experts in Mexico (17–19) and Latin America (20). MAFLD is twice as common in adolescents with type 2 diabetes than in those without and is associated with insulin resistance (21, 22). At present, patients with DM2 and MAFLD are regarded as having higher morbidity and cardiovascular risk (23).

HDLs are lipoproteins considered to be atheroprotective that reduce the risk of CVD (24); several epidemiological studies have evidenced that low plasma concentrations of high-density lipoprotein cholesterol (HDL-C) are an independent risk factor for CVD (25). However, current evidence suggests that the physicochemical properties (26) and functional capacity (27) of HDLs may be more important than HDL-C levels in the prediction of CHD and may play a key role in the pathogenesis of atherosclerosis (28).

The first “protective effect” of HDLs that was reported was reverse cholesterol transport (RCT), a physiological process involving multiple components through which cholesterol from peripheral tissues is transported by HDL particles to the liver, where it is excreted in the bile (29, 30). Moreover, HDLs have key biological atheroprotective properties, such as cholesterol efflux capacity (CEC), and antioxidant, anti-inflammatory, antithrombotic, and immunomodulating activities (31, 32). Finally, HDLs also inhibit LDL-cholesterol particle oxidation through the paraoxonase enzyme activity (an antioxidant protein associated with HDL) (24). CEC is the ability of HDL to accept cholesterol from macrophages, one of the most important steps in RCT (30). Macrophage CEC, a measure of HDL function, is strongly and inversely associated with carotid intima–media thickness and CVD likelihood, regardless of HDL-C levels (33). At present, CEC is considered a new biomarker that describes a key step in RCT, which in turn is inversely associated with CVD incidence (34, 35).

It is known that the static measurement of serum HDL-C levels currently performed in clinical practice may not adequately capture the antiatherogenic properties of the highly heterogeneous HDL particles (33). Only a few studies have established a relationship between different biomarkers associated with HDL, and none of them have directly compared their associations with cardiovascular events (34, 35).

Previous studies have shown a decrease in CEC among adult patients with DM2 as opposed to healthy individuals (36); however, this condition has not been assessed in pediatric patients with DM2. Our research group had previously reported changes in the distribution of HDL subpopulations and in the HDL particle lipid composition among adolescents with DM2 and MAFLD (37). However, whether HDL function is altered or not still needs to be determined in order to show if those physicochemical abnormalities are associated with HDL CEC alteration, with a subsequent deterioration of the atheroprotective mechanisms of HDLs and an increase in the risk of CVD among adolescents with DM2. Consequently, the purpose of this study was to assess the impact of MAFLD on CEC in adolescents with DM2.

## 2 Materials and methods

### 2.1 Subjects

This cross-sectional study included 70 adolescents, 47 of whom had DM2 and were 10–18 years old, both male and female, in stages II–V based on Tanner's scale. They attended the outpatient clinic service for children with diabetes in Hospital Infantil de México Federico Gómez (Mexico City, Mexico). The control group included the remaining 23 adolescents. All patients with diabetes were diagnosed according to the American Diabetes Association criteria (38), based on the International Society for Pediatric and Adolescent Diabetes guidelines (9), with progression time of at least 6 months. Exclusion criteria were: evidence of thyroid dysfunction, kidney disease, liver disease other than MAFLD, chronic or acute infections and therapy with lipid-regulating drugs (over 6 months before the study). Control subjects were recruited from a public high school in Mexico City. This group included normoglycemic subjects, with no clinical evidence of diabetes or thyroid, kidney, or liver disease, and no MAFLD (PDFF < 6.5%). They were not receiving vitamin supplements, nor were they following a specific diet at the time of the study. Written consent and assent from parents and teenagers, respectively, were obtained in all cases. The protocol was approved by the Institutional Ethics and Research Committee of Hospital Infantil de México Federico Gómez (HIM-2018-061. SSA 1500) and followed the principles of the Declaration of Helsinki.

Based on a previous study by Apro et al. (36), the sample size was estimated to identify CEC differences with 80% power and 5% significance for a required sample size of 23 subjects in each group.

### 2.2 Methodology

All participants had to answer a questionnaire, and their weight (kg), height (m), waist circumference (cm), and arterial pressure (AP) were measured. AP was measured through auscultation. Systolic and diastolic arterial pressure (SAP and DAP) were assessed after the subjects had remained seated for at least 10 min, using the correct bracelet size for each participant. Three readings were recorded for each individual, using the average value between the second and third reading for the analysis. Waist circumference was measured with a fiberglass tape, which cannot be easily stretched, up to the nearest 0.5 cm at the middle point between the lower part of the rib cage and the upper part of the iliac crest following a normal exhalation (39). BMI was estimated by dividing subjects' weight in kg by the square of their height. Puberty status was assessed through a physical examination performed by an experienced pediatric endocrinologist using Tanner's classification (40).

Blood samples were collected in two vacutainer tubes with anticoagulant (ethylenediaminetetraacetic acid) following 12-h fasting. Samples were placed on ice and transported to the lipids laboratory of the Endocrinology Department of Instituto Nacional de Cardiología Ignacio Chávez, where the tubes were centrifuged at 4°C 2,500 rpm for 20 min. Determination of both lipid (total cholesterol, triglycerides, and HDL-C) and glucose profiles, as well as blood chemistry tests, were performed in a COBAS c311 automatic analyzer, using commercial enzymatic-colorimetric kits (Roche Diagnostics, Mannheim, Germany). Low-density lipoprotein cholesterol (LDL-C) was estimated using Friedewald's formula modified by De Long (41). Apolipoprotein B (ApoB) and ApoA-I were quantified by immunoturbidimetric assay in a COBAS c311 automatic analyzer with commercial kits (Roche Diagnostics, Mannheim Germany). The reproducibility and accuracy of lipid and lipoprotein assessments were periodically tested by the Lipids Standardization Program from the Center for Disease Control and Prevention (LSP-CDC, Atlanta, GA. USA). Intra- and inter-assay variation of coefficients are below 3% for all measurements. An immunonephelometry assay was used to determine ApoB and ApoA using a BN Pro Spec nephelometer (Dade Behring Marburg GmbH, Schwalbach, Germany) according to the manufacturer's method. Inter-assay coefficients of variation were below 6%. In the DM2 group, A1c hemoglobin levels (HbA1c) were measured by high-performance liquid chromatography.

### 2.3 HDL subtypes

Total plasma HDLs were isolated from samples stored at −70°C by sequential ultracentrifugation at 1.21 g/ml density at 10°C in a Beckman TL-100 ultracentrifuge. Resulting total HDLs were dialyzed using phosphate buffer (pH 7.4) and loaded into 4%–25% polyacrylamide gel to perform polyacrylamide gel electrophoresis. Gel proteins were stained with Coomassie brilliant blue R-250, and the gels were then scanned and digitalized using a GS-670 Bio-Rad densitometer. Molecular AnalystTM software was used for their analysis. For each gel, migration distance intervals were obtained through the estimation of a standard curve for stable high molecular weight proteins (thyroglobulin 12 nm; ferritin 12.2 nm; catalase 10.4 nm; lactate-dehydrogenase 8.2 nm and albumin 7.1 nm) based on their relative migration distance. Relative proportions of each HDL subtype were estimated based on the following size intervals: HDL3c 7.9–8.45 nm; HDL3b 8.45–8.98 nm; HDL3a 8.98–8.94 nm; HDL2a 8.94–10.58 nm; and HDL2b 10.58–12.36 nm (42, 43). Each subtype presented a variation coefficient below 10%. The average HDL particle size represents the overall distribution of HDL subtypes and was estimated as the average size for each HDL subtype interval (nm) multiplied by its relative area on densitometric scanning. In this case, the variation coefficient was lower than 1%.

The amount of total protein, total cholesterol (TC), free cholesterol (FC), phospholipids (PL), and triglycerides (TG) in isolated HDLs was determined using commercially available enzymatic assays in a COBAS c311 analyzer. Cholesteryl esters (CE) were estimated by multiplying the difference between TC and FC by 1.67 (44). The total lipoprotein mass was estimated as the sum of total proteins, CE, FC, PL and TG.

### 2.4 Cholesterol efflux capacity

Cholesterol efflux was determined using J774 mouse macrophages according to the procedure described by Hafiane and Genest (45) with slight modifications. Briefly, cells were labeled for 24 h in the presence of an acyl-CoA:cholesterol acyltransferase inhibitor (SANDOZ, 2 μg/mL, Sigma-Aldrich CAS number 78934-83-5). This was done using 0.5 mL/well of 1 μCi/mL [1,2-3H] cholesterol (Perkin Elmer) in RPMI plus 1% fetal bovine serum. To stimulate ABCA1 expression (46), J774 cells were incubated for 18 h with medium containing 0.3 mM cAMP (Sigma-Aldrich CAS number 93882-12-3) and 0.2% bovine serum albumin in RPMI. The values at time zero were obtained from cell wells harvested before adding the patient serum sample. The percentage of 3H-cholesterol released by cells is calculated using the following formula: (c.p.m. in medium/c.p.m. time zero) × 100. Cholesterol efflux refers to the fraction of total cellular cholesterol released in 6 h to apoB-depleted serum sample (20 μg of apo A1/mL) added to each well. All samples were processed in triplicate on the same plate. A control sample was processed in each assay, to adjust the results and reduce inter-test variability, which was less than 10%.

### 2.5 Metabolic-associated fatty liver disease

Quantification of fat deposits in the liver was performed through magnetic resonance spectroscopy to determine the presence of MAFLD, using the Stimulated Echo Acquisition Mode (STEAM) sequence, a multi-echo sequence using 90°—90°—90° pulses, with higher sensitivity for liver fat quantification (47, 48). Analyses were performed by means of a single-voxel technique (2 × 2 × 2 cm). Liver fat was quantified by adding the area under the spectral curve of liver lipids (0.9–3.0 ppm). Water appears as a single peak at 4.7 ppm and fat as multiple peaks due to the presence of different chemical bonds between protons and adjacent atoms in fat. The values obtained were expressed as fat fraction, which is the fat percentage of the overall voxel (water and fats): Fat fraction = fats/(fats + water) × 100 (47). MAFLD was diagnosed when fat fraction ≥6.5% was detected (49). This study has 90% sensitivity and 87% specificity for MAFLD diagnosis (47, 49). This type of study was chosen because it does not involve radiation and allows for the objective assessment of fatty liver by using a quantitative PDFF index, allowing for the identification of different degrees of disease (50). We support the diagnosis of MAFLD, following the diagnostic criteria for pediatric MAFLD proposed by Eslam M et al. (16).

### 2.6 Data analysis

Data are expressed as mean ± standard deviation or median (maximum and minimum values) based on data distribution. The three groups were compared using a variance analysis (ANOVA) or Kruskall–Wallis test. A correlation analysis was performed to assess the relationship between PDFF and HDL distribution and composition, and the relationship between PDFF and CEC. All analyses were performed with SPSS V20 statistical software. All *p* values ≤0.05 were considered statistically significant.

## 3 Results

In order to assess the effect of MAFLD on CEC, patients with DM2 were categorized into two groups based on the absence or presence of liver fat (fat fraction <6.5% or >6.5%, respectively). The total group with diabetes included 47 adolescents with DM2 with a mean age of 15 years, 35 of which were female (74.5%). The DM2 non-MAFLD group and DM2 with MAFLD group included 16 and 31 subjects, respectively. The control group consisted of 23 subjects, none of whom had MAFLD (all reported a PDFF value <6.5%). Table 1 shows a comparison of the clinical, biochemical, and anthropometric characteristics of patients with DM2 and non-diabetic subjects based on the presence or absence of MAFLD. Non-diabetic adolescents were younger as opposed to the DM2 and MAFLD patient group, with a lower Tanner stage. BMI (*p* < 0.001) and waist circumference (*p* < 0.001) values, as well as glucose and ApoB concentrations, were higher in DM2 groups than in the non-DM2 groups. DAP and Gamma-glutamyltransferase (GGT) concentrations were higher in the DM2 and MAFLD group as opposed to the non-diabetic group. DM2 duration, HbA1c levels, and triglyceride concentrations were higher in the DM2 with MAFLD group as opposed to the DM2 non-MAFLD group. HDL-C values were lower in both DM2 groups as opposed to the non-diabetic group (*p* < 0.001). No differences in SAP or TC, ApoA, creatinine, Aspartate transaminase (AST), Alanine transaminase (ALT), Gamma-glutamyltransferase (GGT), Alkaline phosphatase (ALP) plasma concentrations were observed among the three groups.

**Table 1:** Clinical, biochemical, and anthropometric characteristics in non-diabetic adolescents (control group) and DM2 adolescents according to MAFLD score.

	Control *n* = 23	DM2_		*P* value
		Without	With	
		MAFLD	MAFLD	
		*n* = 16	*n* = 31	
Sex (M/F)	11/12	6/10	6/25	0.080^§^
Age (years)	13.5 ± 2.5	15.1 ± 1.8	15.9 ± 1.6***	**<0.001** *
Weight (kg)	51.7 ± 10.5	66.6 ± 15.3**	64.6 ± 14.4***	**<0.001** *
Size (m)	1.58 ± 0.08	1.64 ± 0.12	1.58 ± 0.08	0.106*
BMI (gg/m^2^)	20.3 ± 2.9	24.1 ± 3.4**	25.6 ± 5.0***	**<0.001** *
BMI (*z* score)	0.4 (−1.9 to −1.7)	1.1 (−1.2 to −2.1)**	1.2 (−1.0 to −4.1)***	**0.015** ^†^
Waist (cm)	71.2 ± 6.8	84.0 ± 10.5**	86.6 ± 13.7***	**<0.001** *
Waist/size index	0.44 (0.37− 0.50)	0.48 (0.40−0.61)**	0.66 (0.43−0.81)***	**<0.001** ^†^
SAP (mmHg)	103.9 ± 6.0	102.5 ± 10.6	105.2 ± 9.0	0.617*
DAP (mmHg)	63.1 ± 3.1	65.5 ± 9.0	68.8 ± 7.3***	**0.011** *
Tanner 3–5 (%)	74.00	88.00	96.00	**0.046** ^§^
Progression (months)	N/A	40.0 ± 24.1	54.6 ± 28.6	**0.001** ^‡^
HbA1c (%)	N/D	7.3 ± 1.0*^a^*	8.2 ± 2.2	**0.032** ^b^
Glucose (mg/dl)	80.6 ± 4.4	190.0 ± 121.6**	203.0 ± 97.0***	**<0.001** *
Total cholesterol (mg/dl)	152.1 ± 20.4	168.7 ± 36.7	171.2 ± 45.5	0.157*
Triglycerides (mg/dl)	86.4 (43−184)	139.6 (47−317)	189.5 (55−575)***	**0.001** ^†^
HDL-c (mg/dl)	51.9 ± 10.9	43.3 ± 6.9**^,a^	38.5 ± 9.1***	**<0.001** *
LDL-c (mg/dl)	86.6 ± 18.0	103.0 ± 32.9	102.4 ± 26.9***	**0.005** *
ApoB (mg/dl)	78.5 ± 13.5	99.3 ± 28.1**	107.0 ± 32.2***	**0.001** *
ApoA (mg/dl)	141.6 ± 25.5	136.0 ± 13.8	130.7 ± 19.4	0.160*
Uric acid (mg/dl)	5.3 (3−7.5)	5.5 (2.5−8.6)	4.3 (2.4−8.7)***	**0.037** ^†^
Creatinine (mg/dl)	0.6 (0.4−1.1)	0.6 (0.4−1.0)	0.6 (0.4−0.8)	0.234^†^
AST (U/L)	19.6 (13.2−28.5)	19.7 (9.0−40.5)	24.3 (9.2−115.1)	0.108^†^
ALT (U/L)	14.4 (6.5−30.8)	21.4 (7.1−73.3)	26.4 (3.8−143.4)	0.747^†^
ALP (U/L)	207.7 (57.2 −401.0)	139.6 (56.9−227.3)	132.0 (69.4−377.9)	0.070^†^
GGT (U/L)	16.1 (7.8−39.3)	27.4 (7.0−84.5)	34.0 (8.7–177.7)***	**0.015** ^†^
IGF-1 (ng/ml)	240.6 ± 87.7	223.5 ± 78.6	217.8 ± 40.7	0.630
IGF-1 (*z* score)	1.1 ± 1.3	1.4 ± 1.3	1.4 ± 1.3	0.713
PDFF (%)	4.8 ± 1.0	5.2 ± 1.2**	12.2 ± 7.1***	**<0.001**

BMI, body mass index; Waist, waist circumference; SAP, systolic arterial pressure; DAP, diastolic arterial pressure; AST, aspartate transaminase; ALT, alanine transaminase; ALP, alkaline phosphatas; GGT, gamma-glutamyltransferase; N/A, not applicable; IGF-1, insulin-like growth factor 1; PDFF, proton density fat-fraction.

Data are expressed as mean ± standard deviation (SD), median (minimum and maximum values) and frequencies.

* ANOVA;

** *p* < 0.05 control vs. DM2 without MAFLD;

*** *p* < 0.05 control vs. DM2 with MAFLD (Bonferroni–Dunn's test).

^†^ Kruskal–Wallis test for independent samples.

^‡^ Mann–Whitney *U* test.

^§^ ×2 test.

^a^ *p* < 0.05 DM2 without MAFLD vs. DM2 with MAFLD.

^b^ Student's t-test.

The values in bold indicate statistical significance in the tests performed (*p* < 0.05).

At the time of the study, 100% of DM2 patients had been prescribed biguanide (metformin) therapy, whereas 64% were prescribed insulin therapy in a basal-bolus scheme. Even though no differences were found in insulin use between both DM2 groups, MAFLD patients required higher doses of insulin per weight every day to achieve their glycemic goals than non-MAFLD patients (0.64 ± 0.25 vs. 0.42 ± 0.26 IU/kg/day; *p* = 0.028).

To evaluate the association of the fraction of liver fat with HDL distribution and composition, a simple linear correlation analysis was performed in patients with DM2 (Table 2). There was a significant negative correlation between the percentage of hepatic fat (PDFF) and the ratio of HDL2b (*r*^2^ = −0.233, *P* = 0.026), HDL2a (*r*^2^ = −0.121, *P* = 0.013), and the average particle size (*r*^2^ = −0.190, *P* = 0.009), and a positive association was observed with HDL3c subpopulations (*r*^2^ = 0.188, *P* = 0.011); about composition inside HDL particle, a positive association was observed with% PDFF and the TG (*r*^2^ = 0.257, *P* = 0.007) and negative with CE (*r*^2^ = −0.111, *P* = 0.010), with FC (*r*^2^ = −0.403, *P* = <0.001).

**Table 2:** Correlation analysis between PDFF (%) and both HDL subpopulation distribution and chemical composition in adolescents with DM2.

	Proton density fat-fraction (%) *n* = 47		
	*r*	*Β*	*P value*
HDL subpopulations			
HDL2b (%)	−0.237	−0.233	**0**.**026**
HDL2a (%)	−0.269	−0.121	**0**.**013**
HDL3a (%)	−0.169	−0.118	0.084
HDL3b (%)	0.113	0.022	0.179
HDL 3c (%)	0.276	0.188	**0**.**011**
HDL size (nm)	−0.285	−0.190	**0**.**009**
HDL composition			
Total protein (%)	0.092	0.134	0.227
Phospholipids (%)	0.058	0.264	0.319
Triglycerides (%)	0.299	−0.257	**0**.**007**
Cholesterol esters (%)	−0.281	−0.111	**0**.**010**
Free cholesterol (%)	−0.372	−0.403	**<0**.**001**

Values were estimated using Pearson's correlation analysis.

The values in bold indicate statistical significance in the tests performed (*p* < 0.05).

**Figure 1:**
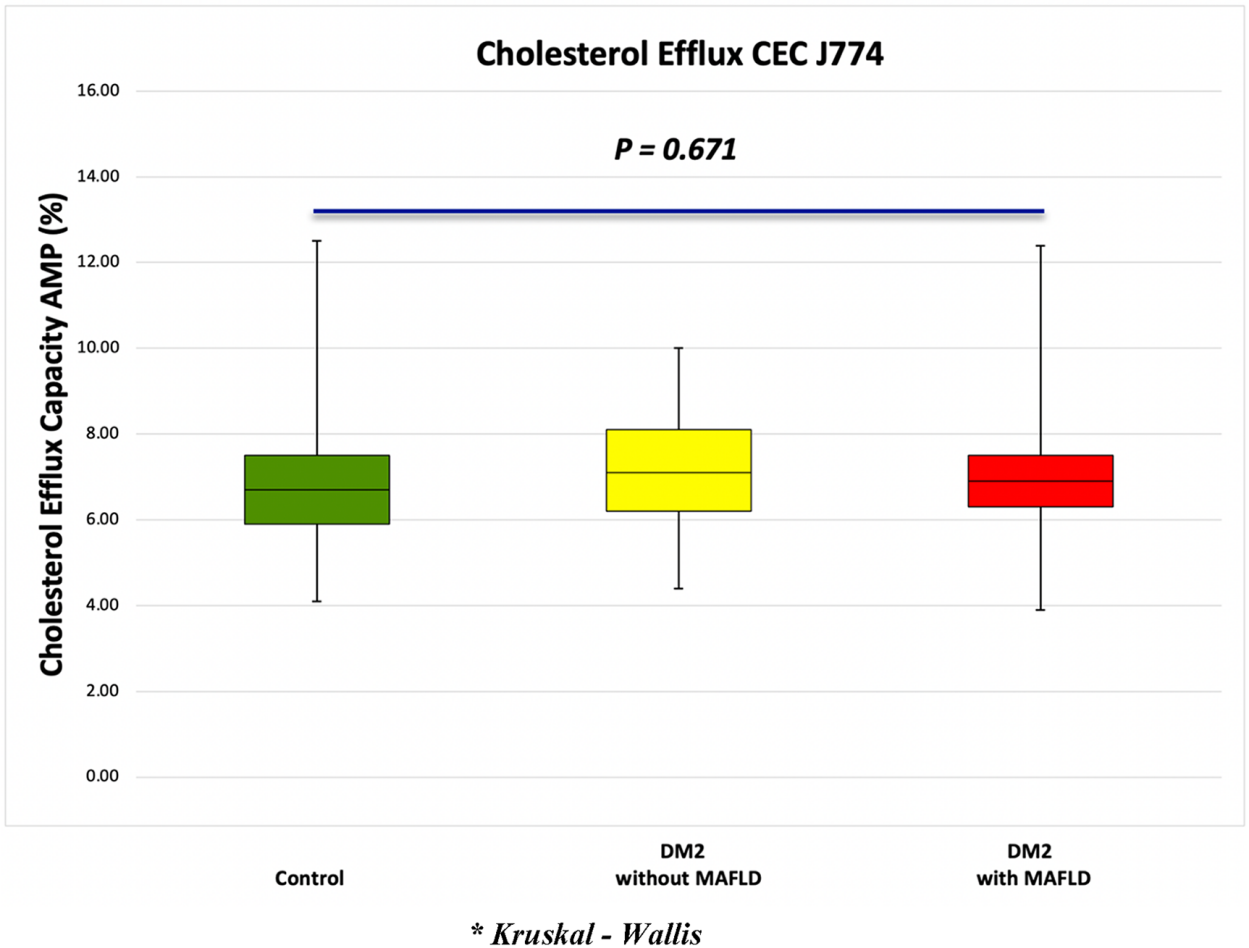
Effect of fatty liver on cholesterol efflux capacity in subjects with DM2.

Regarding HDL functionality, in patients with DM2, the presence of MAFLD had no effect on the functionality of HDL particles (*p* ≦ 0.671). We did not observe differences in the percentage of CEC in subjects with DM2 and MAFLD, compared to the group without MAFLD (6.7% ± 1.8% vs. 7.1% ± 1.7%, *p* ≦ 0.770); similarly, we did not observe differences compared to the control group (7.1% ± 1.7% vs. 6.9% ± 1.6%, *p* ≦ 0.670) (Figure 1). To assess the association between CEC and HDL distribution and composition, a simple linear correlation analysis was performed in patients with DM2 (Table 3). Moreover, a negative correlation between CEC and the amount of triglycerides (TG) within particles was observed (*r*^2^ = −0.320, *P* = 0.007), along with a positive and significant correlation between CEC and the amount of cholesterol esters (*r*^2^ = 0.269, *P* = 0.05), within the particle.

**Table 3:** Correlation analysis between cholesterol efflux (%) and both HDL subpopulation distribution and chemical composition in adolescents with DM2.

	Cholesterol efflux CEC J774 (%) *n* = 47		
	*r*	*Β*	*P value*
HDL subpopulations			
HDL2b (%)	0.033	0.070	0.783
HDL2a (%)	0.044	0.062	0.715
HDL3a (%)	0.090	0.092	0.461
HDL3b (%)	−0.093	−0.125	0.445
HDL 3c (%)	−0.041	−0.061	0.733
HDL size (nm)	0.070	0.117	0.563
HDL composition			
Total protein (%)	0.062	0.012	0.084
Phospholipids (%)	−0.011	−0.033	0.220
Triglycerides (%)	−0.319	−0.320	**0**.**007**
Cholesterol esters (%)	0.231	0.269	**0**.**050**
Free cholesterol (%)	−0.207	−0.105	0.086

Values were estimated using Rho of Spearman correlation analysis.

The values in bold indicate statistical significance in the tests performed (*p* < 0.05).

## 4 Discussion

Our study shows that in this group of adolescents with DM2, the presence of MAFLD is not associated with CEC, a key measure of HDL functionality; however, it is associated with abnormalities in the distribution and lipid composition of HDL particles. In this study, MAFLD prevalence in adolescents with DM2 is similar to the one reported in other studies: 66% vs. 60%–80% (11–13, 16). High IR is not the only explanation for the high MAFLD prevalence observed in adolescents with DM2 (21, 22); recent studies have reported that MAFLD is associated with inadequate glycemic control and contributes to chronic complications such as CVD (51). Our study yielded similar results because adolescents with DM2 and MAFLD had higher HbA1c levels than adolescents with DM2 but without MAFLD. Moreover, insulin requirements were higher among patients with DM2 and MAFLD. These findings are vital because an inadequate metabolic control and the presence of comorbidities at an early age could be associated with reduced life expectancy and increased morbidity during adulthood. In terms of metabolic profile, adolescents with DM2 had lower HDL-C levels than non-diabetic adolescents. Of the three groups, the DM2 and MAFLD group showed the lowest levels of HDL-C, along with higher levels of triglycerides, LDL-C, and Apo B as opposed to non-diabetic adolescents. Based on these differences, this group may be considered as one with a higher atherogenic risk and will require more aggressive therapies to avoid complications at earlier ages.

Previous studies have reported a decrease in CEC among adult subjects with NAFLD (52, 53). An association between liver fat deposits and alterations in the physicochemical characteristics of HDLs has been reported in adolescents with NAFLD (54), as well as alterations in HDL structure and composition in adolescents with DM2 (55). HDL structure and composition dramatically change in certain inflammatory states; this pro-atherogenic phenotype shows strong correlation with the content of the lipids within the liver (56), which clearly suggests a correlation between DM2, MAFLD, and CVD.

In this group of patients, we could not observe differences in CEC in our DM2 population compared to that of adolescents without DM2, nor in those who preselected some degree of MAFLD. However, current studies have revealed that the substrate required for ABCA1-mediated (i.e., ApoA-I and preβ1-HDL) CEC was significantly reduced in patients with NAFLD, which suggests that ApoA-I and preβ1-HDL are the cause of the deterioration observed in HDL CEC (57). Moreover, Fadaei et al. concluded that J774 CEC alteration treated with cAMP is an independent risk factor for higher occurrence of subclinical atherosclerosis, defined as an increase in the carotid intima–media thickness (cIMT) and the presence of atherosclerotic plaque in patients with MAFLD.

The results found in this group of adolescents with DM2 and MAFLD, where we initially found a slight elevation of the CEC in adolescents with DM2, and later a subtle decrease in the CEC in those with DM2 and MAFLD. This could explain a probable metabolic phenomenon, since the physicochemical composition and distribution of the particles is associated with the percentage of liver fat. Our results show that the chemical composition of HDL particles (triglycerides and cholesterol esters) is related to the CEC of HDL in young patients with DM2, which may have future implications because this change in HDL function has been associated with the prevalence of coronary heart disease and recurrent events in patients with DM2 (33–35, 57).

In agreement with previous studies in adults with DM2, we observed a shift towards the smaller HDL3 subclass and a reduction in the larger HDL2 subclass in adolescents with DM2, which is associated with increased cardiovascular risk (58). In the adult population, DM2 and obesity have been shown to profoundly affect HDL metabolism and lead to changes in HDL composition and a shift towards small HDL3 particles (58, 59), as well as marked changes in cholesteryl ester transfer protein (CETP) and lecithin–cholesterol acyltransferase (LCAT) activities (59). Although in our study we did not measure these enzymatic activities, we believe that in future studies this would help to elucidate the specific pathways involved in the observed changes in HDL composition and functionality, which may increase cardiovascular risk in children with DM2.

## 5 Study limitations and strengths

Our study has some potential limitations. First, because of its cross-sectional nature, we cannot draw a causal relationship between MAFLD and HDL function. Second, the biological relevance of these findings in terms of MAFLD progression is currently unknown because our study did not assess the association between CEC percentages and MAFLD severity (steatosis only, steatohepatitis, fibrosis) to establish a correlation with the severity of the disease. However, the significance of these findings lies in the fact that ours is the first study performed in a pediatric population with DM2 and MAFLD that assessed the functional capacity of HDLs by measuring CEC.

Based on our findings, we don't observe differences in the CEC, but we found that chemical composition of HDL particles (triglycerides and cholesterol esters) is related to the CEC of HDL in young patients with DM2; and the percentage of liver fat is associated with abnormalities in the distribution and lipid composition of HDL particles. These may be associated with a decrease in the atheroprotective capacity of HDLs, which increases the risk for CVD in patients with DM2 and MAFLD.

A limitation, given the cross-sectional nature of our study, was that nutritional factors were not assessed in this group of patients; however, given the growing scientific evidence that dietary intervention and a balanced diet have a positive impact on HDL metabolism and functionality in adult individuals with type 2 diabetes, we consider it would be valuable for future research to carry out such an assessment.

## 6 Conclusions

In conclusion, in this group of adolescents with DM2, the presence of MAFLD was not associated with CEC; however, it is associated with abnormalities in the distribution and lipid composition of HDL particles. The momentum generated by the original proposal for MAFLD in the adult population and following the recommendations for pediatric MAFLD will be a step forward in helping to study the impact of MAFLD on the atheroprotective properties of HDL in the pediatric population.

## Data Availability

The raw data supporting the conclusions of this article will be made available by the authors, without undue reservation.
